# A Protocol for Modeling Human Bone Inflammation: Co-Culture of Osteoblasts and Osteoclasts Exposed to Different Inflammatory Microenvironments

**DOI:** 10.3390/mps8050097

**Published:** 2025-09-01

**Authors:** Araceli Valverde, Afsar Raza Naqvi

**Affiliations:** 1 Department of Periodontics, College of Dentistry, University of Illinois Chicago, Chicago, IL 60612, USA; afsarraz@uic.edu; 2 Department of Microbiology and Immunology, University of Illinois Chicago, Chicago, IL 60612, USA

**Keywords:** osteoblasts, osteoclasts, bone metabolism, inflammation, periodontitis, 2D cell co-culture

## Abstract

Bone remodeling relies on the coordinated activity of osteoblasts (OBs) and osteoclasts (OCs). Disruptions in OB-OC balance can lead to diseases such as periodontitis, a chronic microbial-induced inflammatory disease. To investigate how inflammation affects OB-OC interactions, we standardized an in vitro 2D indirect co-culture system using primary human OB and OC precursors from peripheral blood mononuclear cells in a transwell setup, which allows paracrine signaling and separate analysis of each cell type. When exposed to bacterial lipopolysaccharides (*Aa LPS* and *E. coli LPS*) and proinflammatory cytokines (IL-6 and TNF-α), we observed that inflammatory stimuli significantly increased OC differentiation, particularly TNF-α, while *E. coli LPS* specifically suppressed OB activity as observed by the expression of key markers and cellular staining. These results demonstrate that microbial and host-derived inflammatory factors can differentially modulate bone cell behavior. This approach offers a physiologically relevant and ethically advantageous alternative to animal models to screen dual-targeted bone therapies to restore perturbed metabolism.

## 1. Introduction

Bone remodeling is a precisely controlled physiological process involving the coordinated activities of osteoblasts (OBs), which drive bone formation, and osteoclasts (OCs), which mediate bone resorption. This interplay between OBs and OCs is crucial for preserving skeletal integrity and homeostasis [1,2,3,4]. Disruption of OB-OC communication can lead to bone-related diseases such as osteoporosis and periodontitis [5,6,7,8]. There is a critical need to develop advanced models for bone disorders to better understand the complex cellular and molecular mechanisms underlying skeletal diseases. A comprehensive investigation of disease mechanisms will facilitate the evaluation of novel therapeutic strategies in a controlled and translationally relevant manner.

Conventional preclinical models, particularly those utilizing animal subjects, frequently exhibit limited translational applicability due to interspecies physiological disparities, substantial financial burdens, and restricted experimental throughput [9,10]. Conversely, in vitro co-culture systems employing human-derived cells provide a more physiologically relevant and ethically advantageous platform [11,12,13,14,15]. The development of in vitro OB-OC co-culture systems that mimic cellular interactions under physiological conditions has become essential for advancing our understanding of bone biology and for testing new therapeutic approaches [11,12,13,14,15]. Particularly, 2D indirect co-culture systems, where OBs and OCs are maintained in discrete compartments yet communicate via shared soluble factors, have gained prominence as robust and insightful methodologies [11,15]. These platforms facilitate detailed analyses of paracrine signaling, cellular differentiation, and the modulatory effects of biomaterials, endogenous factors, or pharmacological agents on bone cell function [11,15].

Periodontitis (PD) is a chronic, multifactorial inflammatory disorder marked by the progressive degradation of the tooth-supporting tissues, notably the periodontal ligament and alveolar bone [8,16,17,18]. The pathogenesis of PD is orchestrated by a complex interplay between dysbiotic microbial communities within dental biofilms and the host immune response, culminating in persistent inflammation and subsequent destruction of connective tissue and bone [8,16,17,18]. A hallmark of PD is the perturbation of the tightly regulated coupling between OB and OC, which are responsible for bone metabolism [1,2,3,4]. Pro-inflammatory cytokines and bacterial virulence factors potentiate osteoclastogenesis while concurrently suppressing osteoblastic activity, thereby shifting the bone remodeling equilibrium toward net bone loss [16,17,18]. This dysregulation accelerates alveolar bone resorption, undermining tooth stability and ultimately resulting in tooth loss if left unaddressed [16,17,18]. Elucidating the molecular and cellular mechanisms governing OB and OC dysfunction in PD is imperative for the development of targeted therapeutic interventions aimed at preserving alveolar bone integrity and promoting oral health.

In this study, we present a rigorously standardized in vitro protocol for the 2D indirect co-culture of human primary OB, isolated from trabecular bone, and OC precursors, derived from human peripheral blood mononuclear cells (PBMCs). Employing a transwell system, this model enables bidirectional paracrine communication between OB and OC while permitting discrete analysis of each cellular population. To validate the applicability of this platform, we systematically assessed the effects of oral (*Aggregatibacter actinomycetemcomitans*) and non-oral (*Escherichia coli*) bacteria-derived lipopolysaccharide LPS (*Aa LPS* and *E. coli LPS*) and proinflammatory cytokines (interleukin-6 [IL-6] and tumor necrosis factor-alpha [TNF-α]) on the phenotypic and functional responses of both OB and OC under an inflammatory microenvironment.

The system described herein establishes a robust and reproducible in vitro platform for the preclinical assessment of dual-targeted therapeutic agents designed to simultaneously modulate OB and OC activity. This approach is particularly pertinent for the restoration of alveolar bone homeostasis in pathologies such as PD, where dysregulation of OB-OC coupling underlies disease progression. By facilitating precise interrogation of OB-OC crosstalk under defined inflammatory and microbial conditions, this model advances translational bone research and supports the rational development of innovative therapeutic strategies aimed at mitigating bone loss and promoting tissue regeneration.

## 2. Experimental Design

**Figure 1:**
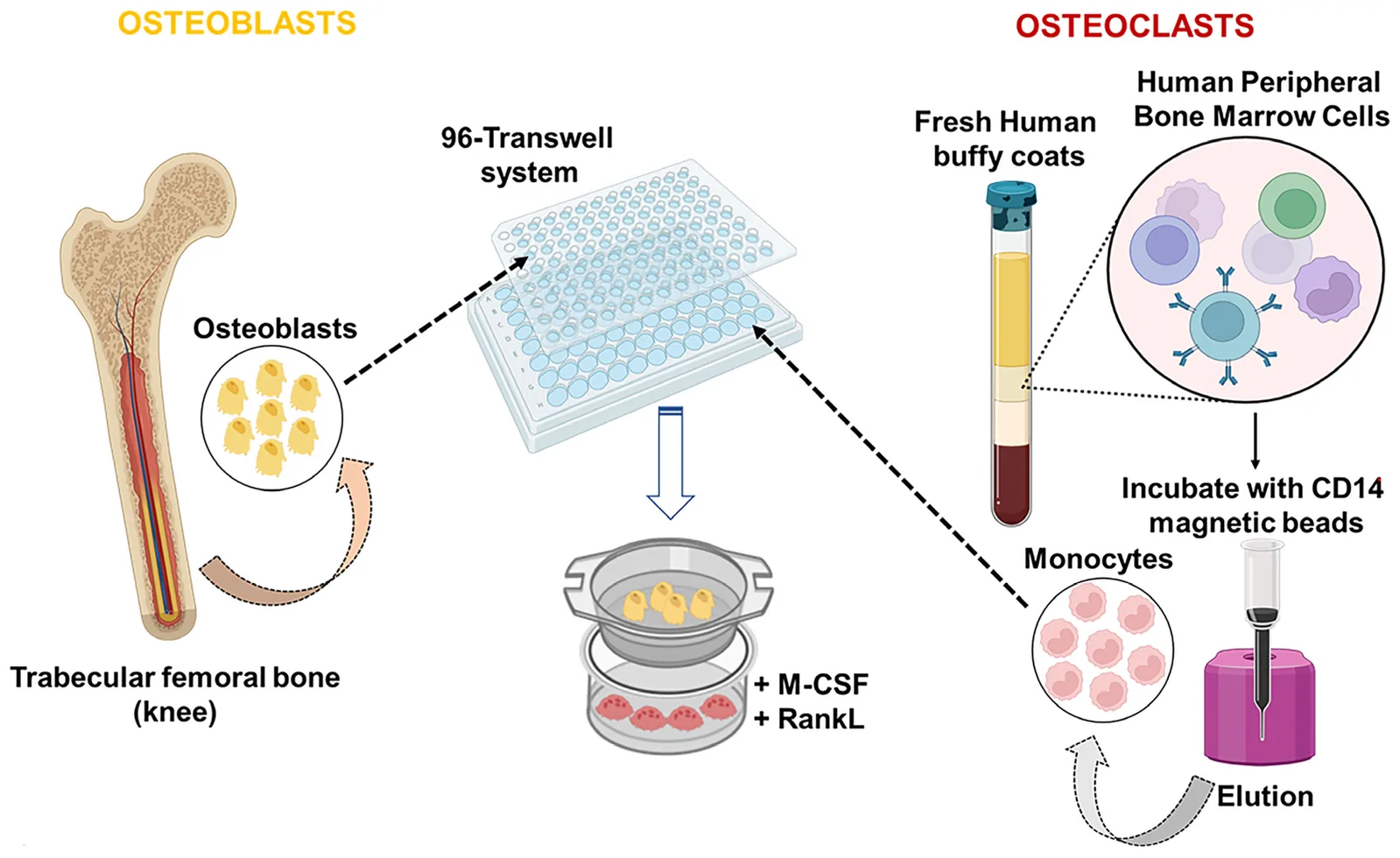
Schematic representation of the indirect co-culture workflow.

**Figure 2:**
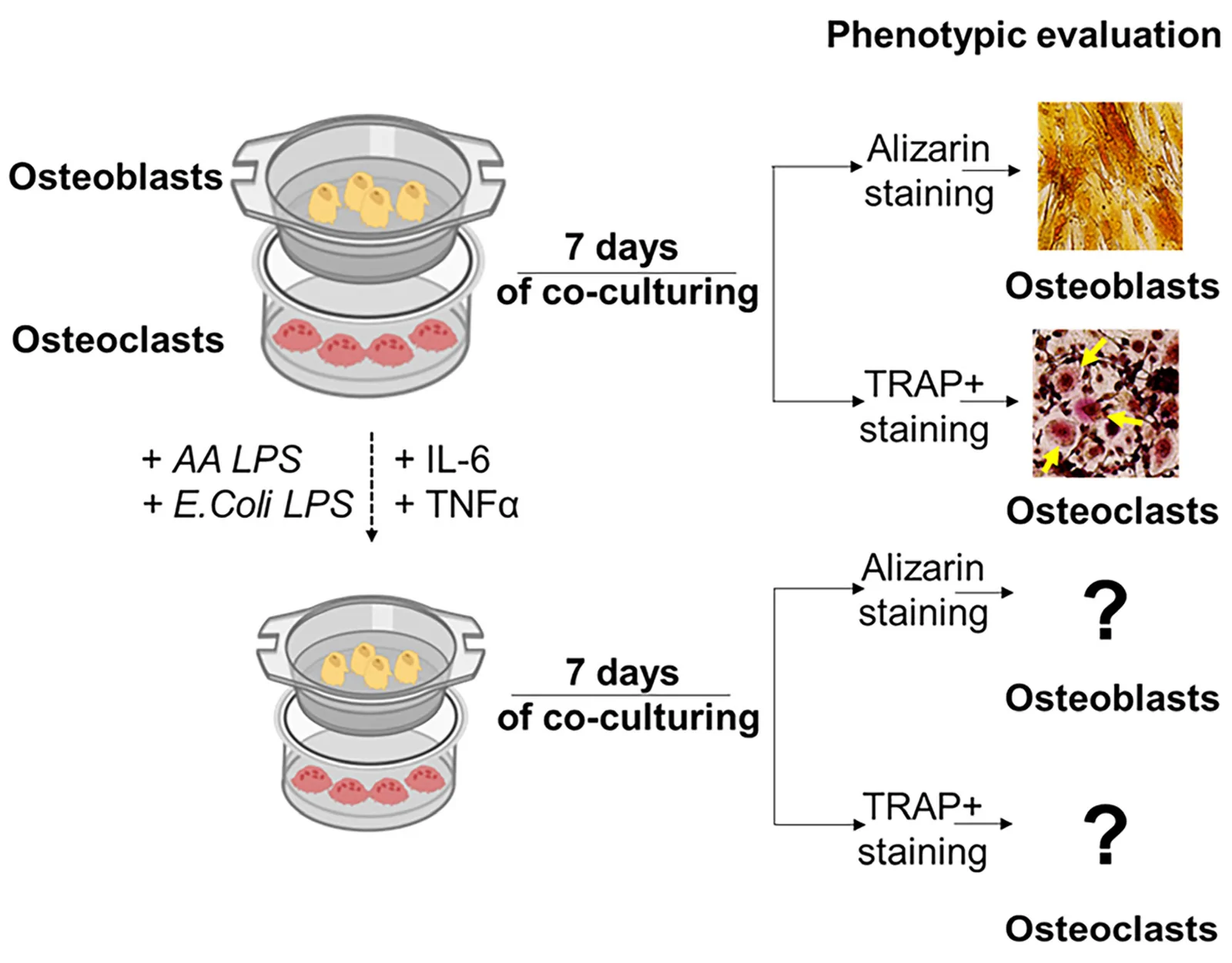
Phenotypic characterization of osteoblasts and osteoclasts under inflammatory microenvironments. Yellow arrows indicate TRAP+ osteoclasts.

This protocol describes a sophisticated 2D indirect co-culture system utilizing primary human OB and OC to replicate the cellular interactions that characterize the bone microenvironment in vitro. The experimental workflow is systematically outlined, with estimated timeframes for each procedural step depicted in the accompanying schematic diagrams. Figure 1 details the methodology for establishing the OB-OC indirect co-culture, while Figure 2 illustrates the assessment of cellular phenotypes through Alizarin Red staining to quantify OB activity and tartrate-resistant acid phosphatase (TRAP) staining to evaluate OC activity (Figure 2).

CD14+ monocytes were seeded in the lower compartment and differentiated into osteoclasts over a 5-day period using macrophage colony-stimulating factor (M-CSF) and receptor activator of nuclear factor kappa-B ligand (RANKL). Concurrently, primary human osteoblasts (OBs) were seeded into the upper transwell insert and cultured to confluence (approximately 5 days). Following this, the transwell insert was positioned above the material-containing well to initiate a 7-day indirect co-culture, thereby facilitating paracrine signaling between OBs and OCs.

After establishing the indirect co-culture by positioning the transwell insert above the OC-containing well, cells were treated with 50 ng/mL of *A. actinomycetemcomitans* lipopolysaccharide (*Aa LPS*), 50 ng/mL of *E. coli* lipopolysaccharide (*E. coli LPS*), and 10 ng/mL each of interleukin-6 (IL-6) and tumor necrosis factor-alpha (TNF-α) for 7 days to simulate inflammatory conditions. At the end of the co-culture period, OBs were assessed for activity using Alizarin Red staining, while OCs were evaluated for functional activity via tartrate-resistant acid phosphatase (TRAP) staining. The expression of key osteoblast and osteoclast gene markers was quantified by RT-qPCR. The effects of inflammatory stimuli on cell viability were evaluated using MTT assays.

### 2.1. Materials

- Primary Human Osteoblasts (Promocell GmbH, Heidelberg, Germany, Cat. No.: C-12720)
- SupplementMix/Osteoblast Growth Medium (Promocell GmbH, Heidelberg, Germany, Cat. No.: C-39615)
- Buffy Coat (Our Blood Institute, Rantoul, IL, USA, Cat. No.: BC-8L)
- Cytica Ficoll-Paque™ Premium, 1.085 g/mL (Fisher Scientific Company LLC, Pittsburgh, PA, USA, Cat. No.: 45-001-755)
- DMEM (Dulbecco’s Modified Eagle’s Medium) (Corning, Manassas, VA, USA, Cat. No.: 10-013-CV)
- CD14 MicroBeads, human (Miltenyi Biotec, Bergisch Gladbach, Germany, Cat. No.: 130-050-201)
- Human TRANCE (RANKL) (soluble), Animal-Free Recombinant Protein (PeproTech, Secaucus, NJ, USA, Cat. No.: AF-310-01-10UG)
- Human M-CSF, Animal-Free Recombinant Protein (PeproTech, Secaucus, NJ, USA, Cat. No.: AF-300-25-10UG)
- Lipopolysaccharides from *Escherichia coli* O55:B5 (Sigma-Aldrich, Saint Louis, MO, USA, Cat. No.: L2880-10MG)
- *Aa LPS* was kindly provided by Dr. Keith Kirkwood (Department of Oral Biology, School of Dental Medicine, University at Buffalo, Buffalo, NY, USA) and characterized previously [19]
- Human IL-6 Recombinant Protein, PeproTech^®^ (PeproTech, Secaucus, NJ, USA, Cat. No.: 200-06)
- Human TNF-alpha Recombinant Protein, PeproTech^®^ (Peproech, Secaucus, NJ, USA, Cat. No.: 300-01A)
- Phosphate-Buffered Saline, 1× without calcium and magnesium, pH 7.4 ± 0.1 (Corning, Manassas, VA, USA, Cat. No.: 21-040-CM)
- Fetal Bovine Serum (Life Technologies Corporation, Carlsbard, CA, USA, Cat. No.: 26140-079)
- L-Glutamine 200 mM (100X) (Life Technologies Corporation, Carlsbard, CA, USA, Cat. No.: 25030-081)
- Penicillin Streptomycin Solution, 100× (Corning, Manassas, VA, USA, Cat. No.: 30-002-Cl)
- Trypsin-EDTA (0.25%), phenol red (Life Technologies Corporation, Carlsbard, CA, USA, Cat. No.: 25200056)
- UltraPure™ 0.5 M EDTA, pH 8.0 (Invitrogen, Grand Island, NY, USA, Cat. No.: 15575-038)
- ACK Lysing Buffer (Life Technologies Corporation, Carlsbard, CA, USA, Cat. No.: A10492-01)
- Dimethyl sulfoxide (Sigma-Aldrich, Saint Louis, MO, USA, Cat. No.: D8418)
- Trypan Blue Solution, 0.4% (Life Technologies Corporation, Carlsbard, CA, USA, Cat. No.: 15250061)
- Molecular Biology Grade Water (Corning, Manassas, VA, USA, Cat. No.: 46-000-Cl)
- Ethanol, Absolute (200 Proof), Molecular Biology Grade, Fisher BioReagents™ (ThermoFisher Scientific, Fair Lawn, NJ, USA, Cat. No.: BP2818-500)
- 2% Alizarin Red Stain (Lifeline Cell Technology, Frederick, MD, USA, Cat. No.: CM-0058)
- Leukocyte Acid Phosphatase (TRAP) Kit (Sigma-Aldrich, Saint Louis, MO, USA, Cat. No.: 387A-1KT)
- CellTiter 96 AQueous Cell Proliferation Assay Kit (Promega, Madison, WI, USA, Cat. No.: G3580)
- Invitrogen™ TRIzol™ Reagent (Invitrogen, Grand Island, NY, USA, Cat. No.: 15-596-026)
- miRNeasy Kit for miRNA Purification (Qiagen, Gaithersburg, MD, US, Cat. No.: 217084)
- High-capacity cDNA Reverse transcription kit (Life Technologies Corporation, Carlsbard, CA, USA, Cat. No.: 4374967)
- PowerUp™ SYBR™ Green Master Mix for qPCR (Life Technologies Corporation, Carlsbard, CA, USA, Cat. No.: A25743)

### 2.2. Equipment

- Cell Incubator (NuAire, Horsham, PA, USA, Cat. No.: NU-5810)
- Flow laminar cabinet (ThermoFisher Scientific, Fair Lawn, NJ, USA, Cat. No.: 1307)
- Cellometer Auto 1000 (Nexcelom Bioscience, Lawrence, MA, USA, Cat. No.: 9943)
- EVOS Fluorescence Microscope (EVOS, ThermoFisher Scientific, Fair Lawn, NJ, USA, Cat. No.: AMF4300)
- StepOne 7500 thermocycler (Life Technologies Corporation, Carlsbard, CA, USA, Cat. No.: 4376373)
- Eppendorf High Capacity Refrigerated (Eppendorf North America, Inc., Enfield, CT, USA, Cat. No.: 5810R)
- T75 Cell Culture Flask, Vented, Sterile, 100/CS (Thomas Scientific, Swedesboro, NJ, USA, Cat. No.: 21A00M453)
- 15 mL Centrifuge Tubes, Polypropylene, 50/Tray, 500/CS (Thomas Scientific, Swedesboro, NJ, USA, Cat. No.: 1159M36)
- 50 mL Centrifuge Tubes, Polypropylene, 25/tray, 500/CS (Thomas Scientific, Swedesboro, NJ, USA, Cat. No.: 1158R10)
- Corning^®^ HTS Transwell^®^ 96-well permeable supports (Sigma-Aldrich, Milwaukee, WI, USA, Cat. No.: CSL3381-1EA)
- LS Columns (Miltenyi Biotec, Bergisch Gladbach, Germany Cat. No.: 130-042-401)
- MidiMACS™ Starting Kit (LS) (Miltenyi Biotec, Bergisch Gladbach, Germany Cat. No.: 130-042-301)

### 2.3. Cell Viability Assay

Cell viability assays were performed using the CellTiter 96 AQueous Cell Proliferation Assay Kit according to the manufacturer’s protocol. Briefly, OB were seeded in the transwell insert and treated with *Aa LPS* and *E. coli LPS* at a concentration of 50 ng/mL, along with individual cytokines IL-6 and TNF-α at a concentration of 10 ng/mL for 7 days. Following the treatment period, OB seeded in the transwell insert were separated, and the cell viability assay was assessed by directly adding 20 μL of AQueous One Solution (MTS) to each well. Cells were incubated for 1–3 h at 37 °C, protected from light, until adequate color developed. The absorbance was measured at 490 nm. Blank-subtracted values were normalized to controls to calculate percent viability.

### 2.4. Total RNA Isolation, cDNA Synthesis, and Quantitative PCR

Following the treatment period, the transwell insert (containing OB) was separated from the lower compartment (containing OC). Cells were washed three times with PBS, and 700 µL of TriZol reagent (Invitrogen, Waltham, MA, USA) was added to a 96-well culture plate for each condition. Total RNA was extracted using the miRNeasy Micro Kit, and 250 ng of RNA was reverse-transcribed with the High-Capacity cDNA Reverse Transcription Kit. RT-qPCR was performed on a StepOne 7500 using SYBR Green Master Mix to assess RUNX2 and Cathepsin K (CTSK), with β-actin as the housekeeping gene (primer sequences are listed in Table S1). Relative expression was calculated using the 2^−ΔΔCt^ method from triplicate Ct values.

### 2.5. Statistical Analysis

Data were analyzed using GraphPad Prism (Version 10.4.2; LaJolla, San Diego, CA, USA). The results are represented as standard deviation or ±SEM from three independent replicates, and experiments were conducted at least three times. All datasets were assessed for normality prior to statistical analysis by performing a Shapiro-Wilk test, and then a parametric test (one-way ANOVA or unpaired two-tailed *t*-test) was performed. *p* < 0.05 was considered significant.

## 3. Procedure

### 3.1. Peripheral Blood Mononuclear Cell Isolation Procedure

(Clean the hood area with 70% ethanol before working and switch the gloves at any time).

Isolate peripheral blood mononuclear cells (PBMCs) from buffy coats of healthy donors following the Ficoll-Paque density-gradient method.

**Caution**: Blood collection and all laboratory procedures were conducted under Institutional Biosafety Committee (IBC) approval, with protocols adhering to biosafety levels and pathogen-handling requirements specified by the approved IBC protocol (24-099). Informed consent must be obtained from patients.

1. Prepare 1× PBS solution containing 2 mM EDTA. Add 4 mL EDTA to 1 L of 1× PBS.
2. Label 6 tubes/donor (50 mL centrifuge tubes) and add 20 mL of Ficoll-Hypaque.
3. Fill a T75 cell culture flask with 150 mL of 1× PBS-EDTA solution (described above).

**CRITICAL STEP:** How to open the blood bag: make a brief cut in the blood bag harboring the plastic bag, and then cut the longest protruding tubes filled with blood using a sterile surgical blade and discard the plastic tube in the paper bag. Discard the blade in a biohazard-labeled container.

4. Dilute the buffy coat sample with an equal amount of 1× PBS (1:1 final dilution).
5. Gently pour 30 mL of the diluted buffy coat overlaying the Ficoll layer, being careful not to break the gradient layer.
6. Gradient centrifugation: centrifuge the tubes at 1329 rpm (RCF 300) for 30 min with breaks off to enable the PBMCs to stay on the gradient layer without being forced into the erythrocyte layer. Maintain the centrifuge temperature at 4 °C and keep the brakes off.
7. At the end of the first gradient centrifugation, PBMCs form a “ring” between Ficoll and plasma, as illustrated in Figure 1 Harvest each layer of PBMCs and transfer it into 3 new 50 mL tubes/donor. Then, add up to 50 mL of PBS + 5% FBS (500 μL/L of PBS).

**CRITICAL STEP:** Harvest the PBMCs ring with a Pasteur pipette.

8. Centrifuge the tubes at RT and 1500 rpm (RCF 382) for 10 min.
9. Discard the supernatant and harvest each PBMC pellet and transfer it into 3 new 15 mL tubes. Then, add up to 15 mL of PBS + 5% FBS.
10. Centrifuge the tubes at RT and 1500 rpm (RCF 382) for 5 min.
11. Discard the supernatant and resuspend the PBMC pellet in 1 mL ACK lysing buffer and transfer it into 2 new 15 mL tubes. Then, add up to 15 mL of ACK lysing buffer.

**Note:** ACK Lysing Buffer is a solution used to selectively lyse (break open) red blood cells, allowing for the isolation and study of white blood cells (such as lymphocytes) from blood samples.

12. Centrifuge the tubes at RT and 1500 rpm (RCF 382) for 5 min.
13. Discard the supernatant and resuspend the PBMC pellet in 1 mL of PBS + 5% FBS and transfer it into 1 new 15 mL tube.
14. Repeat steps 12 and 13.
15. PBMCs are now ready for CD14+ sorting using the MidiMACS system.

### 3.2. Sorting of CD14+ from PBMCs Using the MidiMACS System and Osteoclast Culture

1. Place the LS columns in the MidiMACS magnetic stand and add 4 mL of PBS + 5% FBS to hydrate the sorting columns.
2. Add 120 μL of CD14+ beads to the PBMCs (previous step 15) and incubate at 4 °C for 30 min.
3. Before starting the sorting, add up to 4 mL of PBS + 5% FBS to the PBMCs.
4. Load 4 mL (1 mL at a time) in a column. CD14- monocytes will pass through the column, while CD14+ monocytes remain stuck in the column.
5. For elution of the CD14+ monocytes, remove the columns from the MidiMACS and add 4 mL of PBS + 5% FBS. Use a plunger provided by the manufacturer to elute the CD14+ monocytes into new 15 mL tubes.
6. Centrifuge the tubes at RT and 1500 rpm (RCF 382) for 5 min, keeping the brakes on.
7. Discard the supernatant and resuspend the CD14+ pellet in 10 mL of an incomplete DMEM media.
8. Seed the cells at the bottom of the 96 transwell at a density of 50,000 cells/well.
9. After 2 h, replace the media with complete DMEM media containing 50 ng/mL of Monocyte Colony Stimulant Factor (MSCF) and 50 ng/mL of Receptor Activator of NF-κB Ligand (RANKL).
10. Refresh the complete DMEM media plus the components MSCF and RANKL at the concentrations described above every 2 days for 5 days.

### 3.3. Osteoblast Culture

Primary human OB were obtained from PromoCell, isolated from knee and/or femoral head tissue in accordance with the manufacturer’s protocols. Immediately post-isolation, OBs are cryopreserved in liquid nitrogen to ensure cellular viability. Each cryovial contains over 500,000 viable cells upon recovery. Thawing and initial seeding of these cells corresponds to passage 2.

1. Fill a 50 mL tube with 50 mL of complete PromoCell Growth Medium. Place the tube in an incubator (37 °C, 5% CO_2_) for 30 min.
2. Remove the cryovial from the liquid nitrogen container and immediately immerse the vial in a water bath (37 °C) up to the height of the screw cap for 2 min. Ensure that no water enters the thread of the screw cap.
3. Thoroughly rinse the cryovial with 70% ethanol under a laminar flow bench. Open the vial and transfer the cells to a 15 mL tube containing the prewarmed medium from step 1.
4. Centrifuge the tubes at RT and 1500 rpm (RCF 382) for 5 min.
5. Discard supernatant and resuspend the cell pellet with 1 mL of prewarmed medium from step 1 and place the resuspended cells into the T75 cell culture flask. Add up to 10 mL of prewarmed medium from step 1.
6. Place the T75 cell culture flask containing the OB in an incubator (37 °C, 5% CO_2_) for cell attachment. Replace the medium every two days. The cells should be subcultured once they have reached 70–90% confluency.
7. Aspirate the medium from the T75 culture cell flask, wash with 5 mL of PBS, and add 2 mL of Trypsin/EDTA for a few seconds.
8. Remove the Trysin/EDTA and place the T75 cell culture flask in an incubator for cell detachment for 5 min.
9. Collect the OB with 10 mL of complete medium to neutralize the leftover Trypsin/EDTA and centrifuge the tubes at RT and 1500 rpm (RCF 382) for 5 min.
10. Discard the supernatant and resuspend the cell pellet with 1 mL of fresh complete media and count the cells using a Cellometer.
11. OB were plated on the transwell insert at a density of 2500 cells/well. Replace every two days for 5 days.

### 3.4. Assembly of OB/CD14+ Indirect Co-Culture and Phenotypic Evaluation of OB and OC Treated with Aa LPS, E. coli LPS, IL-6, and TNF-α

After 5 days of separate culture, OB and CD14+monocyte-derived OC were assembled into a 2D indirect co-culture system using a transwell insert. OB were cultured in the upper transwell, while OC were seeded on the material surface in the lower compartment. Two days post-assembly, the indirect co-culture was treated with *Aa LPS* and *E. coli LPS*, at a concentration of 50 ng/mL, and with individual cytokines IL-6 and TNF-α at 10 ng/mL. Treatments were maintained for 7 days.

Following the treatment period, the transwell insert (containing OB) was separated from the lower compartment (containing OC), and phenotypic evaluation was performed using Alizarin Red staining for OB activity and TRAP staining for OC activity.

To evaluate the OB phenotype, we used the Alizarin red staining:

1. Remove the medium and wash the cells with PBS.
2. Fix the cells with absolute ethanol for 30 min at room temperature. Ethanol was removed, and wells were air-dried.
3. 2% Alizarin Red S solution was added per well and incubated for 15 min. Wells were rinsed three times with distilled water and allowed to dry before imaging.
4. Air dry and evaluate microscopically.

To evaluate the OC phenotype, we used the tartrate-resistant acid phosphatase (TRAP) staining:

Prepare Fixative Solution by combining 3.1 mL of Citrate Solution, 8.1 mL of acetone, and 1 mL of 37% formaldehyde.

1. Remove the medium and wash the cells with PBS.
2. Fix the OC with Fixative Solution for 30 s. Rinse thoroughly in deionized water: Do not allow slides to dry.
3. Prepare 2 test tubes and add 0.111 mL Fast Garnet GBC Base Solution and 0.111 mL Sodium Nitrite Solution. Mix by gentle inversion for 30 s.
4. Prepare 2 test tubes (A and B) and add:
  Tubes A and B:
    - 10 mL of deionized water prewarmed to 37 °C
    - 0.222 mL of Diazotized Fast Garnet GCB Solution (Step 3)
    - 0.111 mL of Naphthol AS-Bl Phosphate Solution
    - 0.444 mL of Acetate Solution
    - 0.222 mL of Tartrate Solution (Only in tube B)
5. Incubate for 1 h in 37 °C protected from light.
6. Rinse the wells in deionized water, then counterstain for 2 min in Hematoxylin Solution, Gill No. 3.
7. Rinse for several minutes in alkaline tap water to blue nuclei.
8. Air dry and evaluate microscopically.

## 4. Expected Results

### Enhanced Osteoclast Differentiation and Activity Driven by Periodontal Pathogens and Proinflammatory Cytokines

**Figure 3:**
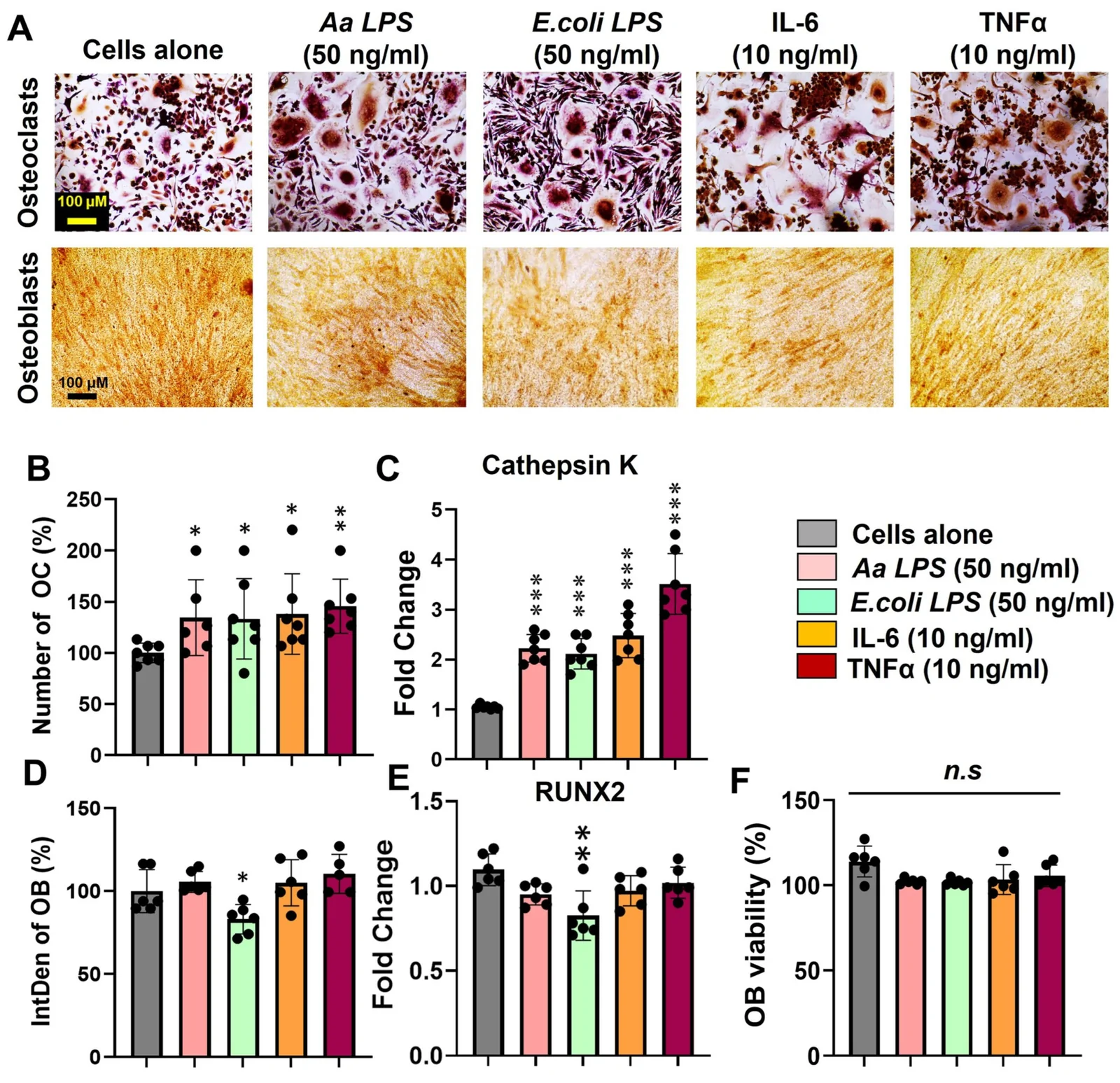
Periodontal pathogens and inflammatory cytokines promote the osteogenic activation in a 2D indirect co-culture assay of OB and OC. Primary human CD14+ monocyte-derived OC and primary human OB were co-cultured in the presence of M-CSF and RANKL for 7 days. Cells were treated with *Aa LPS* (50 ng/mL), *E. coli LPS* (50 ng/mL), IL-6 (10 ng/mL), and TNFα (10 ng/mL) for another 7 days. (**A**) Representative images showing OC differentiation by TRAP staining (**upper panel**) and OB differentiation by Alizarin Red staining (**lower panel**) at day 14 under different proinflammatory conditions. Images were acquired using a 20× objective lens; the scale bar corresponds to 100 μm. (**B**) Bar graphs showing percentages of multinucleated OC under different inflammatory conditions. The total number of OC was normalized to the control group. Each dot represents an individual data point overlaid on the bar graph. (**C**) Quantitative expression of Cathepsin K (CTSK) was examined by RT-qPCR. Actin was used as a housekeeping control. Data are means ± SEM of three independent donors. The Ct values of three replicates were analyzed to calculate fold change using the 2^−ΔΔCt^ method. (**D**) Bar graphs showing quantification of Alizarin Red staining using Integrated density analysis (ImageJ 1.54d software) to assess the OB activity. (**E**) Expression analysis of RUNX2, a key OB marker, by RT-qPCR. Actin was used as a housekeeping control. Data are means ± SEM of three independent donors. The Ct values of three replicates were analyzed to calculate fold change using the 2^−ΔΔCt^ method. (**F**) Cell viability of OB at day 14 cultured under the above-mentioned inflammatory conditions. ANOVA was conducted to calculate *p*-values. * *p* < 0.05, ** *p* < 0.01, *** *p* < 0.001 vs. control untreated. n.s: non-significant.

To elucidate the impact of periodontal pathogens and proinflammatory cytokines on bone cell dynamics, OC and OB responses were systematically evaluated within a 2D indirect co-culture system under defined inflammatory conditions. OC and OB were exposed to *A. actinomycetemcomitans* lipopolysaccharide (*Aa LPS*, 50 ng/mL), *E. coli LPS* (*E. coli LPS*, 50 ng/mL), interleukin-6 (IL-6, 10 ng/mL), and tumor necrosis factor-alpha (TNF-α, 10 ng/mL), with untreated cultures serving as controls. Morphological assessment (Figure 3A, upper panel) revealed a marked increase in OC size and a higher number of multinucleated cells across all treatment groups, with TNF-α treatment eliciting the most pronounced effect. Quantitative analysis revealed that compared with control, *Aa LPS* and *E. coli LPS* elevated OC numbers by 35%, IL-6 augmented OC formation by 40%, and TNF-α markedly induced OC differentiation by 45% (Figure 3B). These data indicate that both bacterial components and proinflammatory cytokines robustly stimulate osteoclastogenesis, with TNF-α exerting the most potent pro-resorptive effect.

To confirm a shift toward a bone-resorptive phenotype under inflammatory conditions, we analyzed CTSK expression, a key marker of OC-mediated bone resorption (Figure 3C). Compared to control cells, *Aa LPS*, *E. coli LPS*, and IL-6 induced CTSK expression by ~2-fold, while TNF-α-treated cells show a ~4-fold increase, suggesting a potent enhancement of osteoclastic activity under TNFα-driven inflammatory signaling (Figure 3B).

OB activity, as measured by integrated density (IntDen) of Alizarin Red staining, did not show any significant changes in response to *Aa LPS* (105%), IL-6 (105%), and TNF-α (110%) relative to untreated controls (Figure 3A; lower panel). Interestingly, OB treated with *E. coli LPS* exhibited a significant reduction in activity (~20%) compared to control (Figure 3D), indicative of a suppressive effect on osteoblastic function.

We further examined the impact of various treatments on OB differentiation by examining the expression of RUNX2, a master regulator of OB lineage commitment and maturation. Among the tested stimuli, only *E. coli LPS* significantly downregulated RUNX2 expression (~20% decrease) relative to control levels, indicating impaired differentiation potential. These transcriptional changes are consistent with the reduced functional activity observed in Figure 3D.

To rule out the impact of the inflammatory environment on the viability of differentiated cells, we performed an MTS assay. Compared to the control, we did not observe any significant effect on the viability of OB in all experimental conditions, suggesting that the tested inflammatory stimuli do not exert a cytotoxic effect. Collectively, these findings demonstrate that inflammatory stimuli significantly enhance OC differentiation while having minimal impact on OB activity, except under *E. coli LPS* exposure, while overall viability remains intact. This dual modulation within the indirect co-culture system underscores a mechanistic basis for the bone remodeling imbalance characteristic of inflammatory bone diseases such as periodontitis.

## 5. Strengths and Limitations

Co-culture assays constitute a sophisticated and physiologically relevant platform for recapitulating the intricate host–microbe interactions that underpin PD pathogenesis. Their principal advantage lies in the capacity to simultaneously culture multiple host cell types, such as OB and OC, under diverse proinflammatory conditions, including exposure to periodontal pathogens and cytokines [20]. This approach closely mirrors the inflammatory milieu of periodontal tissues, thereby enhancing the translational reproducibility of in vitro findings [21]. Furthermore, co-culture systems significantly improve the predictive accuracy of drug response studies by capturing the dynamic interplay between microbial determinants and host immune mechanisms, insights that are frequently overlooked in monoculture or animal models [20]. The adaptability of these assays to advanced platforms, such as three-dimensional (3D) organotypic cultures and organ-on-chip technologies, further augments their structural and functional relevance, bridging the gap between in vitro experimentation and clinical application [20,22]. From an ethical and economic perspective, co-culture assays offer substantial benefits, being more cost-effective, scalable, and ethically acceptable than animal models, thus facilitating high-throughput drug screening in early-stage research [21]. Additionally, these models provide a unique framework for investigating the systemic implications of periodontal inflammation, including its potential contributions to comorbidities such as diabetes and cardiovascular disease [22].

The OB/OC co-culture protocol proposed here incorporates exogenous M-CSF and RANKL to drive and maintain osteoclast differentiation and survival, whereas the other models did not include exogenous RANKL in their baseline conditions. In our hands, osteoclasts fail to survive or exhibit sustained resorptive activity without continuous supplementation of RANKL (with M-CSF provided to support precursor proliferation and survival), underscoring a key methodological distinction that affects feasibility, kinetics, and readouts of OC function. This protocol improves reproducibility and sensitivity while maintaining practical throughput, and it leverages primary human cells to enhance biological relevance. Specifically, we designed the workflow to systematically assess inflammatory impacts by incorporating defined exposures to periodontal bacteria (e.g., *A. actinomycetemcomitans LPS*) and inflammatory cytokines (e.g., TNF-α and IL-6). We standardized media changes, cytokine replenishment, and time points and used blinded ImageJ pipelines to improve reproducibility across experiments. Importantly, we use primary human osteoblasts and monocyte-derived osteoclasts from independent donors, which strengthens robustness and translational relevance compared with immortalized lines. While the assay remains compatible with moderate-throughput formats (24–96 wells), the novel integration of inflammatory modulators and dose–response testing is, to our knowledge, a distinctive feature not examined in this depth in prior OB/OC co-culture protocols.

Despite these considerable strengths, co-culture assays are not without limitations. They present notable limitations when compared to in vivo models. For instance, these systems omit systemic physiological inputs, such as endocrine, immune, and neural signals, that regulate bone dynamics and influence disease progression and treatment responses [23,24]. Similarly, periodontal co-culture models face challenges in replicating the dynamic and heterogeneous microenvironment, including microbial diversity, immune cell infiltration, and oxidative stress, which are essential for accurate disease modeling [21]. Both bone and periodontal co-culture systems may not recapitulate the full complexity of their respective tissue architectures that may affect cellular phenotypes and coupling dynamics [25,26]. Cellular complexity is another shared limitation. OB–OC models often exclude key cell types such as osteocytes, endothelial cells, adipocytes, and immune populations, despite their roles in RANKL/OPG signaling, mechanoregulation, and inflammation [27,28,29]. Likewise, periodontal cocultures may incorporate innate immune elements such as macrophages but frequently lack adaptive immune responses and systemic feedback mechanisms [30]. Long-term maintenance of cocultures, especially those involving anaerobic bacteria and immune cells, remains technically challenging due to limited viability [21]. Finally, the use of 2D or simplified matrices in both systems can distort cell morphology, polarity, gene expression, and functional kinetics, leading to discrepancies in drug responses compared to native tissue behavior [31,32]. While advanced platforms such as organ-on-chip technologies offer promise, their implementation is constrained by the need for specialized equipment and expertise, limiting accessibility [22].

Despite these limitations, co-culture systems remain a powerful, scalable platform for hypothesis testing, mechanistic exploration, and preclinical screening, serving as essential complements to in vivo studies.

## 6. Discussion and Conclusions

This study presents a robust and reproducible in vitro indirect co-culture model that mimics the human bone microenvironment by facilitating paracrine interactions between primary human OB and OC [11]. Using a transwell system, we evaluated the effects of periodontal microbial (*Aa LPS*, *E. coli LPS*) and inflammatory (IL-6, TNF-α) stimuli on OB and OC behavior, providing insights into the cellular dynamics underlying bone remodeling in inflammatory conditions such as periodontitis [11].

Our results reveal that all tested inflammatory and microbial stimuli significantly OC differentiation, with TNF-α eliciting the most robust effect. This observation is consistent with prior reports identifying TNF-α as a potent osteoclastogenic cytokine that facilitates bone resorption by upregulating receptor activator of nuclear factor kappa-B ligand (RANKL) expression and enhancing precursor cell fusion [33]. While OC numbers increased across all experimental conditions, OB activity remained largely unaffected, except in cultures exposed to *E. coli LPS*. This selective inhibition suggests a shift in the bone remodeling equilibrium toward resorption and may be attributable to the distinct immunostimulatory properties of *E. coli LPS*, known to impair OB differentiation and mineralization via Toll-Like Receptor-4 (TLR4)-dependent mechanisms [34]. In brief, *E. coli LPS* activates TLR4, a crucial component of the innate immune response that is also expressed on OB [35]. Upon activation by LPS, TLR4-MyD88/TRIF triggers downstream signaling cascades involving NF-κB and MAPK pathways, which subsequently lead to the downregulation of Wnt/β-catenin and BMP-Smad signaling and suppress expression of key osteogenic markers such as RUNX2, alkaline phosphatase (ALP), and osteocalcin (OCN). This molecular shift impairs osteoblast differentiation and mineralization [36]. Additionally, LPS stimulation promotes the production of pro-inflammatory cytokines, including TNF-α and IL-6, further exacerbating the inflammatory environment and contributing to a shift toward bone resorption [37].

The divergent responses of OB and OC to these stimuli underscore the intricate regulatory networks governing bone remodeling under inflammatory stress, highlighting the value of co-culture systems for dissecting these complex cellular interactions [33]. Importantly, the indirect co-culture model employed in this study enabled the concurrent assessment of both anabolic (OB-mediated) and catabolic (OC-mediated) activities, providing a comprehensive perspective on bone cell dynamics that surpasses the informational yield of monoculture systems [11]. In our indirect co-culture assays inflammatory conditions reduced osteoblast mineralization and enhanced osteoclast activity, consistent with indirect models reporting cytokine-driven suppression of OB differentiation (decreased ALPL/RUNX2/OCN) and promotion of osteoclastogenesis via elevated RANKL/OPG ratios and NF-κB/MAPK activation. However, in the future we will compare direct and indirect co-culture assays to evaluate how different treatments affect OB and OC differentiation and activity and highlight treatments where the absence of contact-dependent coupling in indirect systems leads to under- or overestimation of responses.

To evaluate the functional status of each cell type, we used TRAP staining for osteoclasts and Alizarin Red staining for osteoblasts, as these assays align with their distinct roles in bone remodeling. TRAP staining detects functionally active osteoclasts by labeling tartrate-resistant acid phosphatase, an enzyme highly expressed in bone-resorbing cells. In contrast, Alizarin Red binds to calcium deposits formed during osteoblast-driven mineralization, serving as a reliable indicator of bone formation. Together, these methods accurately assess the activity of OB and OC and reflect their specific contributions to bone homeostasis. In addition, we also showed that OB and OC staining correlates with the expression of key differentiation markers. This dual-readout (OB and OC) capability is particularly advantageous for the preclinical evaluation of therapeutic agents designed to restore bone homeostasis by simultaneously modulating OB and OC function [34,38].

In summary, the indirect co-culture system described herein recapitulates critical aspects of the bone microenvironment and offers a physiologically relevant platform for investigating OB-OC interactions under inflammatory conditions. Our findings demonstrate that microbial and cytokine stimuli, especially TNF-α, potentiate osteoclastogenesis, while *E. coli LPS* selectively impairs osteoblastic activity. These insights advance our understanding of the cellular mechanisms underlying bone loss in periodontitis and substantiate the utility of co-culture models in translational bone research [33].

Future investigations should aim to incorporate additional cellular components, such as immune cells, and leverage advanced platforms, including 3D and organ-on-chip technologies, to further enhance model complexity and translational applicability [34]. Extending the duration of co-culture and integrating real-time monitoring approaches may also yield valuable information regarding the temporal dynamics of bone remodeling. Integrating immune cell populations (e.g., macrophages, T cells, or B cells) to model osteoimmunology could further enhance impact and differentiate the platform. While beyond the scope of the current study, we will evaluate this in future work by adding defined immune subsets and cytokine-modulating conditions, with readouts spanning OB/OC function, inflammatory profiling, and mechanistic signaling. Collectively, this model holds significant promise for facilitating the development of dual-targeted therapies that address both bone formation and resorption in inflammatory bone diseases.

## Data Availability

The original contributions presented in this study are included in the article/Supplementary Material. Further inquiries can be directed to the corresponding author.

## Supplementary Materials

The following supporting information can be downloaded at: https://www.mdpi.com/article/10.3390/mps8050097/s1.

## Abbreviations

2D	Two-dimensional
3D	Three-dimensional
*Aa LPS*	*Aggregatibacter actinomycetemcomitans* lipopolysaccharide
ACK	Ammonium-Chloride-Potassium
*E. coli LPS*	*Escherichia coli* lipopolysaccharide
EDTA	Ethylenediaminetetraacetic acid
IL-6	Interleukin-6 (IL-6)
IntDen	Integrated Density
MSCF	Monocyte Colony Stimulant Factor
OB	Osteoblast
OC	Osteoclast
PBMC	Peripheral blood mononuclear cells
PBS	Phosphate-Buffered Saline
RANKL	Receptor Activator of NF-κB Ligand
TNF-α	Tumor necrosis factor-alpha
TLR4	Toll-Like Receptor 4
TRAP	Tartrate-resistant acid phosphatase

## References

[1] Chen X., Wang Z., Duan N., Zhu G., Schwarz E.M., Xie C. (2018). Osteoblast-Osteoclast Interactions. Connect. Tissue Res..

[2] Tamma R., Zallone A. (2012). Osteoblast and osteoclast crosstalks: From OAF to Ephrin. Inflamm. Allergy Drug Argets.

[3] Kim J.M., Lin C., Stavre Z., Greenblatt M.B., Shim J.H. (2020). Osteoblast-Osteoclast Communication and Bone Homeostasis. Cells.

[4] Matsuo K., Irie N. (2008). Osteoclast-osteoblast communication. Arch. Biochem. Biophys..

[5] Schwartz Z., Goultschin J., Dean D.D., Boyan B.D. (1997). Mechanisms of alveolar bone destruction in periodontitis. Periodontology 2000.

[6] Hienz S.A., Paliwal S., Ivanovski S. (2015). Mechanisms of Bone Resorption in Periodontitis. J. Immunol. Res..

[7] Könönen E., Gursoy M., Gursoy U.K. (2019). Periodontitis: A Multifaceted Disease of Tooth-Supporting Tissues. J. Clin. Med..

[8] Valverde A., George A., Nares S., Naqvi A.R. (2024). Emerging Therapeutic Strategies Targeting Bone Signaling Pathways in Periodontitis. J. Periodontal Res..

[9] Mukherjee P., Roy S., Ghosh D., Nandi S.K. (2022). Role of animal models in biomedical research: A review. Lab. Anim. Res..

[10] Marshall L.J., Bailey J., Cassotta M., Herrmann K., Pistollato F. (2023). Poor Translatability of Biomedical Research Using Animals—A Narrative Review. Altern. Lab. Anim..

[11] Sieberath A., Della Bella E., Ferreira A.M., Gentile P., Eglin D., Dalgarno K. (2020). A Comparison of Osteoblast and Osteoclast In Vitro Co-Culture Models and Their Translation for Preclinical Drug Testing Applications. Int. J. Mol. Sci..

[12] Jolly J.J., Chin K.Y., Farhana M.F.N., Alias E., Chua K.H., Hasan W.N.W., Ima-Nirwana S. (2018). Optimization of the Static Human Osteoblast/Osteoclast Co-culture System. Iran. J. Med. Sci..

[13] Borciani G., Montalbano G., Baldini N., Cerqueni G., Vitale-Brovarone C., Ciapetti G. (2020). Co-culture systems of osteoblasts and osteoclasts: Simulating in vitro bone remodeling in regenerative approaches. Acta Biomater..

[14] Steller D., Scheibert A., Sturmheit T., Hakim S.G. (2020). Establishment and validation of an in vitro co-culture model for oral cell lines using human PBMC-derived osteoclasts, osteoblasts, fibroblasts and keratinocytes. Sci. Rep..

[15] Borciani G., Montalbano G., Baldini N., Vitale-Brovarone C., Ciapetti G. (2022). Protocol of Co-Culture of Human Osteoblasts and Osteoclasts to Test Biomaterials for Bone Tissue Engineering. Methods Protoc..

[16] Kinane D.F., Stathopoulou P.G., Papapanou P.N. (2017). Periodontal diseases. Nat. Rev. Dis. Primers.

[17] Nanci A., Bosshardt D.D. (2006). Structure of periodontal tissues in health and disease. Periodontology 2000.

[18] Slots J. (2013). Periodontology: Past, present, perspectives. Periodontology 2000.

[19] Naqvi A.R., Fordham J.B., Khan A., Nares S. (2014). MicroRNAs responsive to Aggregatibacter actinomycetemcomitans and Porphyromonas gingivalis LPS modulate expression of genes regulating innate immunity in human macrophages. Innate Immun..

[20] Makkar H., Sriram G. (2025). Advances in modeling periodontal host-microbe interactions: Insights from organotypic and organ-on-chip systems. Lab Chip.

[21] Mountcastle S.E., Cox S.C., Sammons R.L., Jabbari S., Shelton R.M., Kuehne S.A. (2020). A review of co-culture models to study the oral microenvironment and disease. J. Oral Microbiol..

[22] Wang C., Xu T., Seneviratne C.J., Ong L.J.Y., Zhou Y. (2024). Modelling periodontitis in vitro: Engineering strategies and biofilm model development. Front. Biomater. Sci..

[23] Florencio-Silva R., Sasso G.R., Sasso-Cerri E., Simões M.J., Cerri P.S. (2015). Biology of Bone Tissue: Structure, Function, and Factors That Influence Bone Cells. Biomed Res. Int..

[24] Tsourdi E., Zillikens M.C., Meier C., Body J.J., Gonzalez Rodriguez E., Anastasilakis A.D., Abrahamsen B., McCloskey E., Hofbauer L.C., Guañabens N. (2020). Fracture risk and management of discontinuation of denosumab therapy: A systematic review and position statement by ECTS. J. Clin. Endocrinol. Metab..

[25] Galán-Díez M., Cuesta-Domínguez Á., Kousteni S. (2018). The Bone Marrow Microenvironment in Health and Myeloid Malignancy. Cold Spring Harb. Perspect. Med..

[26] Verdugo-Avello F., Wychowaniec J.K., Villacis-Aguirre C.A., D’Este M., Toledo J.R. (2025). Bone microphysiological models for biomedical research. Lab Chip.

[27] Boyce B.F., Xing L. (2008). Functions of RANKL/RANK/OPG in bone modeling and remodeling. Arch. Biochem. Biophys..

[28] Tatsumi S., Ishii K., Amizuka N., Li M., Kobayashi T., Kohno K., Ito M., Takeshita S., Ikeda K. (2007). Targeted ablation of osteocytes induces osteoporosis with defective mechanotransduction. Cell Metab..

[29] Lorenzo J., Horowitz M., Choi Y. (2008). Osteoimmunology: Interactions of the bone and immune system. Endocr. Rev..

[30] Langer R., Tirrell D.A. (2004). Designing materials for biology and medicine. Nature.

[31] Lutolf M.P., Hubbell J.A. (2005). Synthetic biomaterials as instructive extracellular microenvironments for morphogenesis in tissue engineering. Nat. Biotechnol..

[32] Ham J., Lever L., Fox M., Reagan M.R. (2019). In Vitro 3D Cultures to Reproduce the Bone Marrow Niche. JBMR Plus.

[33] Remmers S.J.A., de Wildt B.W.M., Vis M.A.M., Spaander E.S.R., de Vries R.B.M., Ito K., Hofmann S. (2021). Osteoblast-osteoclast co-cultures: A systematic review and map of available literature. PLoS ONE.

[34] Owen R., Reilly G.C. (2018). In vitro Models of Bone Remodeling and Associated Disorders. Front. Bioeng. Biotechnol..

[35] Alonso-Pérez A., Franco-Trepat E., Guillán-Fresco M., Jorge-Mora A., López V., Pino J., Gualillo O., Gómez R. (2018). Role of Toll-Like Receptor 4 on Osteoblast Metabolism and Function. Front. Physiol..

[36] Komori T. (2019). Regulation of Proliferation, Differentiation and Functions of Osteoblasts by Runx2. Int. J. Mol. Sci..

[37] Chan W.C.W., Tan Z., To M.K.T., Chan D. (2021). Regulation and Role of Transcription Factors in Osteogenesis. Int. J. Mol. Sci..

[38] Valverde A., Naqvi R.A., Chen Y., Moshaverinia A., George A., Shukla D., Martinez G., Chapa G., Nares S., Naqvi A.R. (2025). Herpesvirus Simplex Virus-1 Exploits Inflammation to Infect Periodontal Stem Cells and Disrupt Lineage Commitment. J. Periodontal Res..

