# Supplementary material for: A Protocol for Modeling Human Bone Inflammation: Co-Culture of Osteoblasts and Osteoclasts Exposed to Different Inflammatory Microenvironments
Source: Methods Protoc. 2025 Sep 1;8(5):97. doi: 10.3390/mps8050097 (PMC12452566; doi:10.3390/mps8050097)
Supplement: Supplementary file 1 [file mps-08-00097-s001.zip › mps-3797192-supplementary.pdf]

methods and protocols

MDPI

Protocol

A Protocol for Modeling Human Bone Inflammation: Co-Culture of Osteoblasts and Osteoclasts Exposed to Different Inflammatory Microenvironments

Araceli Valverde and Afsar Raza Naqvi

Table S1.

Gene Name	Forward (5′ to 3′)	Reverse (5′ to 3′)	Species name
**RUNX2**	AAGCTTGATGACTCTAAACC	TCTGTAATCTGACTCTGTCC	Human
**CTSK**	GAGGCTTCTCTTGGTGTCCATAC	TTACTGCGGGAATGAGACAGGG	Human
**Actin**	GACGACATGGAGAAAATCTG	ATGATCTGGGTCATCTTCTC	Human
